# A combination of SOFA score and biomarkers gives a better prediction of septic AKI and in-hospital mortality in critically ill surgical patients: a pilot study

**DOI:** 10.1186/s13017-018-0202-5

**Published:** 2018-09-10

**Authors:** Chao-Wei Lee, Hao-wei Kou, Hong-Shiue Chou, Hsu-huan Chou, Song-Fong Huang, Chih-Hsiang Chang, Chun-Hsing Wu, Ming-Chin Yu, Hsin-I Tsai

**Affiliations:** 1 Department of Surgery, Chang Gung Memorial Hospital, Linkou, Taiwan, Republic of China; 2 College of Medicine, Chang Gung University, Guishan, Taoyuan, Taiwan, Republic of China; 3 Graduate Institute of Clinical Medical Sciences, Chang Gung University, Guishan, Taoyuan, Taiwan, Republic of China; 4 Division of Nephrology, Kidney Research Center, Chang Gung Memorial Hospital, Linkou, Taiwan, Republic of China; 5 Department of Anesthesiology, Chang Gung Memorial Hospital, Linkou, Taiwan, Republic of China

**Keywords:** Sepsis, Acute kidney injury, AKI, Mortality, SOFA score, NGAL, Calprotectin, Critically ill patients, Intensive care unit, Surgical ICU

## Abstract

**Background:**

Sepsis is a syndrome characterized by a constellation of clinical manifestations and a significantly high mortality rate in the surgical intensive care unit (ICU). It is frequently complicated by acute kidney injury (AKI), which, in turn, increases the risk of mortality. Therefore, it is of paramount importance to identify those septic patients at risk for the development of AKI and mortality. The objective of this pilot study was to evaluate several different biomarkers, including NGAL, calprotectin, KIM-1, cystatin C, and GDF-15, along with SOFA scores, in predicting the development of septic AKI and associated in-hospital mortality in critically ill surgical patients.

**Methods:**

Patients admitted to the surgical ICU were prospectively enrolled, having given signed informed consent. Their blood and urine samples were obtained and subjected to enzyme-linked immunosorbent assay (ELISA) to determine the levels of various novel biomarkers. The clinical data and survival outcome were recorded and analyzed.

**Results:**

A total of 33 patients were enrolled in the study. Most patients received surgery prior to ICU admission, with abdominal surgery being the most common type of procedure (27 patients (81.8%)). In the study, 22 patients had a diagnosis of sepsis with varying degrees of AKI, while the remaining 11 were free of sepsis. Statistical analysis demonstrated that in patients with septic AKI versus those without, the following were significantly higher: serum NGAL (447.5 ± 35.7 ng/mL vs. 256.5 ± 31.8 ng/mL, *P* value 0.001), calprotectin (1030.3 ± 298.6 pg/mL vs. 248.1 ± 210.7 pg/mL, *P* value 0.049), urinary NGAL (434.2 ± 31.5 ng/mL vs. 208.3 ± 39.5 ng/mL, *P* value < 0.001), and SOFA score (11.5 ± 1.2 vs. 4.4 ± 0.5, *P* value < 0.001). On the other hand, serum NGAL (428.2 ± 32.3 ng/mL vs. 300.4 ± 44.3 ng/mL, *P* value 0.029) and urinary NGAL (422.3 ± 33.7 ng/mL vs. 230.8 ± 42.2 ng/mL, *P* value 0.001), together with SOFA scores (10.6 ± 1.4 vs. 5.6 ± 0.8, *P* value 0.003), were statistically higher in cases of in-hospital mortality. A combination of serum NGAL, urinary NGAL, and SOFA scores could predict in-hospital mortality with an AUROC of 0.911.

**Conclusions:**

This pilot study demonstrated a promising panel that allows an early diagnosis, high sensitivity, and specificity and a prognostic value for septic AKI and in-hospital mortality in surgical ICU. Further study is warranted to validate our findings.

**Electronic supplementary material:**

The online version of this article (10.1186/s13017-018-0202-5) contains supplementary material, which is available to authorized users.

## Background

Recently, the Third International Consensus Definitions Task Force (Sepsis-3) has proposed new criteria defining sepsis as the presence of infection and an increase in the Sequential Organ Failure Assessment (SOFA) score greater than or equal to 2, which has been associated with an in-hospital mortality as high as 10% [1]. A change in the SOFA score has been found to have high predictive validity and prognostic accuracy for in-hospital mortality in the intensive care unit setting [2, 3]. Sepsis is a known major contributing factor to the development of acute kidney injury (AKI), and the incidence of AKI among critically ill patients can be as high as 67% [4–6]. AKI is a continuum of clinical manifestations ranging from mild, reversible injury to severe, irreversible damage leading to permanent loss of renal function. Sepsis-associated acute kidney injury (or septic AKI) should, therefore, describe a syndrome characterized by Sepsis-3, in addition to the presence of AKI.

Septic AKI arises in approximately 51–64% of patients with sepsis, with a six- to eightfold increase in the risk of in-hospital mortality [7]. The diagnosis of AKI has been made based on the changes of two parameters, serum creatinine (SCr), and urine output. The Acute Dialysis Quality Initiative (ADQI) working party published the RIFLE criteria in 2004, which differentiated three levels of AKI severity (risk, injury, failure) and two outcome stages (loss and end-stage renal disease) [8]. However, such a diagnosis based solely on SCr or on the detection of oliguria may be inadequate, as it may take up to 48 h before a sufficient change in SCr levels becomes detectable [9, 10]. Of late, new biomarkers related to the underlying pathogenesis of AKI, such as neutrophil gelatinase-associated lipocalin (NGAL) [11] and cystatin C [12] have been studied extensively in the diagnosis and prognosis of septic AKI. Calprotectin, composed of two monomers S100A8 and S100A9, is another novel biomarker. It is produced mainly from neutrophils and monocytes in response to ischemic reperfusion injury [13]. Urine calprotectin appeared to have high diagnostic accuracy in differentiating prerenal from intrinsic AKI [14, 15].

Since sepsis and associated conditions represent a significant proportion of complications in the surgical intensive care unit (ICU), it is mandatory to identify those septic patients at risk for the development of AKI and mortality. The objective of the present study was thus to evaluate several different biomarkers, including NGAL, calprotectin, KIM-1, cystatin C, and GDF-15, along with SOFA scores with regard to predicting the development of septic AKI and associated in-hospital mortality in critically ill surgical patients.

## Methods

### Study population

This study was a prospective cohort study performed in a surgical intensive care unit (ICU) of a 3500-bed tertiary center in Taiwan. The surgical ICU is a 10-bed closed unit managed by a surgeon who is also certified to care for ICU patients. The study was approved by the Institutional Review Board of Chang Gung Memorial Hospital (IRB103-2722A3) and conducted according to the guidelines established by the Declaration of Helsinki. Written informed consent was obtained from all participants. The admission criteria to our surgical ICU include the following: (1) major postoperative complications requiring further invasive intervention; (2) complicated gastrointestinal disorders such as life-threatening gastrointestinal bleeding, fulminant hepatic failure, or severe pancreatitis; (3) hepatic failure complicated with multi-organ dysfunction related to liver transplantation; (4) acute postoperative respiratory distress; (5) cardiovascular instability such as shock of any cause; and (6) other clinical presentations deemed acceptable for ICU admission by the attending physician. We have consecutively recruited all patients admitted to the surgical ICU for 7 months spanning from November 2014 to June 2015. On ICU admission, the patients were initially excluded if they were less than 20 years of age and had a history of chronic kidney disease (CKD) for more than 3 months, inflammatory bowel disease, renal transplantation, or a need for a routine dialysis program. Applying the Sepsis-3 criteria or the identification of an infection site and an increase of greater than or equal to two points in the Sequential Organ Failure Assessment (SOFA) score [1], the patients were allocated into a non-septic group and a septic group. A baseline SOFA score of 0 was assumed for all patients. Among the septic group, some patients were further excluded if no consent form was obtained from the patient or next-of-kin, blood/urine specimens were not collected within the first 24 h of the ICU admission, or patients were transferred to our ICU with the initial treatments started in another ICU. The non-septic group was defined as those patients who had no clinical or laboratory evidence of infection. As this was a pilot study, it was predetermined that patients would be assigned to the septic or non-septic groups in a 2:1 ratio.

Upon admission, patients’ demographic information, comorbidities, type of surgery, use of vasopressors, the Acute Physiology and Chronic Health Evaluation (APACHE II), and the Sequential Organ Failure Assessment (SOFA) scores were recorded. SOFA was calculated sequentially based on the worst value of the parameters including partial pressure of oxygen (PaO_2_), fraction of inspired oxygen (FiO_2_), platelet count, bilirubin, mean arterial blood pressure (MAP), Glasgow Coma Scale (GCS), creatinine, and urine output over the preceding 24 h. The status of AKI was also documented and defined according to the risk, injury, failure, loss of kidney function, and end-stage kidney disease (RIFLE) criteria [8], as follows: a percentage decrease of > 25% in the glomerular filtration rate (GFR) or an increase of ≥ 1.5 times in serum creatinine (SCr) level is defined as risk; a > 50% decrease in GFR or ≥ 2 times increase in SCr is defined as injury; a > 75% decrease in GFR or ≥ 3 times increase in SCr is defined as failure; persistent acute renal failure more than 4 weeks is defined as loss; and complete loss of renal function for more than 3 months is considered end-stage renal disease (ESRD). The patients were followed throughout their ICU stay. CKD was defined as a GFR lower than 60 ml/min/1.73m^2^ using the baseline creatinine and the CKD Epidemiology Collaboration equation [16].

### Blood sampling and assays

Blood and urine samples were obtained as soon as possible after patients were admitted to the ICU. Blood samples were centrifuged at 1500*g* for 10 min, while urine samples were centrifuged at 500*g* for 10 min; both were aliquoted and stored at − 80 °C for batch analysis. Serum and urinary gelatinase-associated lipocalin (NGAL), calprotectin, KIM-1, cystatin C, and GDF-15 were measured using an enzyme-linked immunosorbent assay (ELISA) kit (DuoSet ELISA, R&D Systems; Minneapolis, MN, USA) [17–23]. The dilution ratios for NGAL, calprotectin, KIM-1, cystatin C, and GDF-15 were 1:100, 1:1, 1:100, 1:400, and 1:100, respectively. Albumin, creatinine, procalcitonin, lactate, and C-reactive protein (CRP) were analyzed using enzymatic methods on an automated chemical analyzer in the central laboratory of Chang Gung Memorial Hospital.

### Statistical analysis

The continuous variable data were tested for normality distribution using Kolmogorov-Smirnov and Shapiro-Wilk tests and presented as the mean ± standard error of mean (SEM). The independent sample *t* test and the Mann–Whitney *U* test were used for comparison of the study groups. Categorical variables were compared using Fisher’s test or Pearson’s chi-square test and presented as absolute frequency and percentages. Receiver operating characteristic (ROC) curve analysis was performed to determine the performance of biomarker concentration in the prediction of septic AKI and in-hospital mortality. Data analysis was performed using SPSS version 13 (SPSS Inc., Chicago, IL, USA). *P* values less than 0.05 were considered statistically significant.

## Results

### Study population

**Fig. 1:**
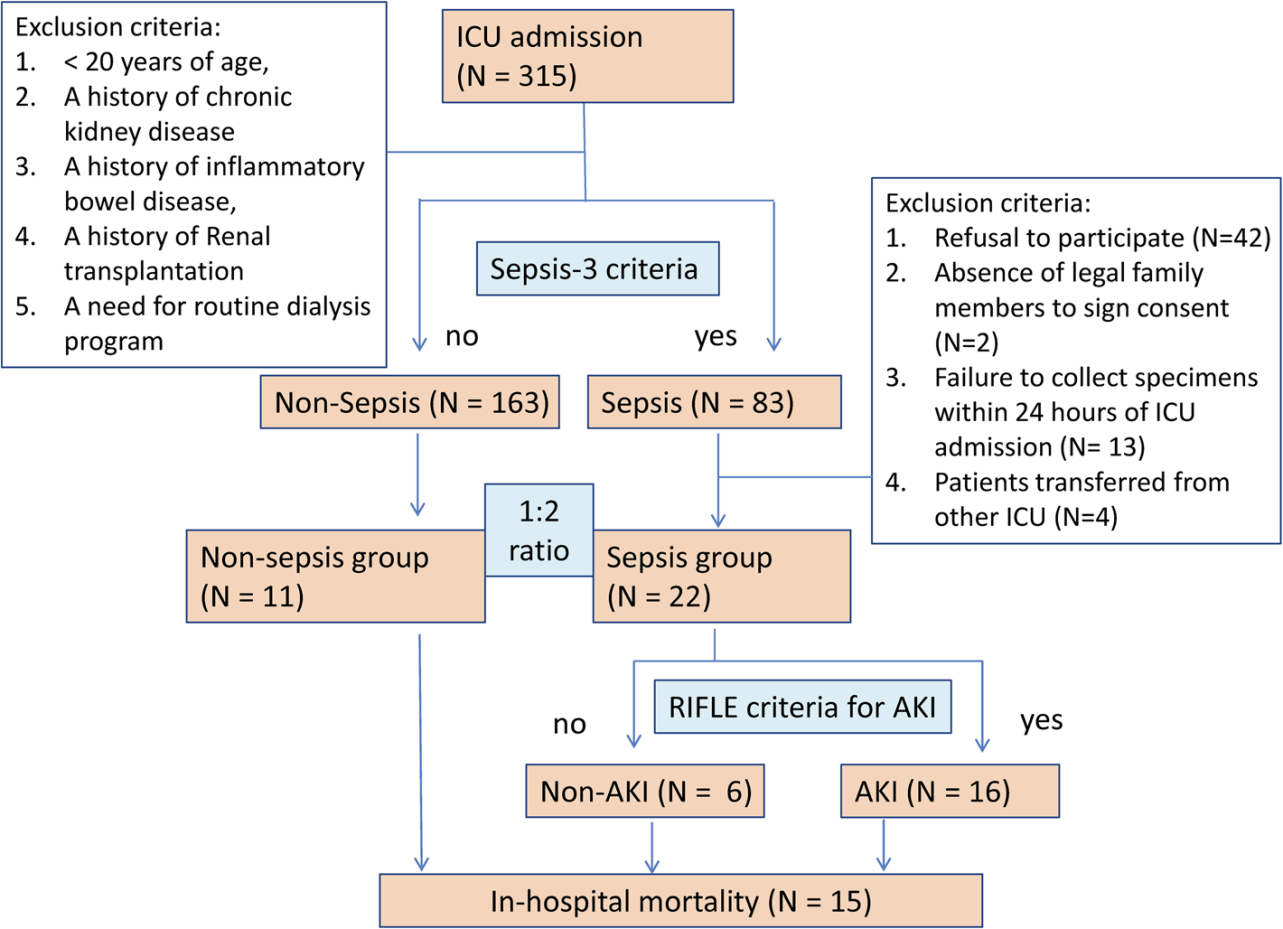
Flow chart of the enrolled patients

Over the 7-month study period, a total of 315 patients were admitted to the surgical ICU, and after applying exclusion criteria, 83 patients were allocated to the septic group, as depicted in Fig. 1. Of the 83 patients with sepsis, 61 were further excluded from the study due to a refusal to participate (*N* = 42), an absence of next-of-kin to consent (*N* = 2), a failure to collect blood/urine specimens within 24 h of ICU admission (*N* = 13), and a transfer from another ICU where initial treatments had been started (*N* = 4). The baseline demographics and clinical characteristics of the 33 patients are shown in Tables 1 and 2. A majority of the patients were males (60.6%) and over 65 years of age (63.6%), with a mean age of 66.3 years. Among these 33 patients, 22 had a diagnosis of sepsis with varying degrees of AKI, while the remaining 11 were free of sepsis. In 15 (45.6%) patients, intra-abdominal infection was the sepsis etiology, 2 patients (6.1%) had pneumonia, 2 (6.1%) had Fournier’s gangrene identified as soft tissue infection, 1 (3.0%) had a urinary tract infection, while 2 (6.1%) had sepsis of unknown origin. Of the 16 patients finally diagnosed with AKI, 14 (42.4%) had a status of R, I, or F, according to the RIFLE criteria, while the other two had a status of L. As depicted in Tables 1 and 2, although 60% of the patients were not on vasopressors, 84.8% of patients required the assistance of mechanical ventilation, with an average of 8.9 ± 20.3 days before successful endotracheal extubation. The length of hospital stay ranged from 2 to 114 days (mean 39.1 ± 25.1 days), with a mean ICU stay of 12.6 ± 25.3 days.

**Table 1:** Demographic characteristics at ICU admission (categorical variable)

Variables		No.	(%)
Age (years)	≥ 65	21	63.6
	< 65	12	36.4
Gender	Male	20	60.6
	Female	13	39.4
BMI (kg/m2)	< 18.5	4	12.1
	18.5–24.9	16	48.5
	25–29.9	12	36.4
	30–34.9	1	3.0
Comorbidity	Diabetes mellitus	5	15.1
	Hypertension	10	30.3
	Cerebrovascular disease	3	9.1
	Cardiovascular disease	5	15.2
	Chronic lung disease	3	9.1
	Liver cirrhosis	4	12.1
	Malignancy	18	54.5
Type of surgery	Hepatobiliary surgery	6	18.2
	Gastrointestinal surgery	21	63.6
	Others	2	6.1
	Without surgery	4	12.1
Sepsis	Yes	22	66.7
	No	11	33.3
Sepsis etiology	IAI	15	45.6
	Pneumonia	2	6.1
	Soft tissue infection	2	6.1
	Urinary tract infection	1	3.0
	Unknown	2	6.1
SOFA score	0–1	1	3.0
	2–7	17	51.5
	8–11	7	21.2
	> 11	8	24.2
Kidney dysfunction (RIFLE criteria)	Normal	17	51.5
	R, I, F	14	42.4
	L, E	2	6.1
Albumin (g/dL)	≥ 3.5	3	9.1
	< 3.5	30	90.9
Use of vasopressors	Yes	13	39.4
	No	20	60.6
Use of MV	Yes	28	84.8
	No	5	15.2

*ICU* intensive care unit, *BMI* body mass index, *SOFA* Sequential Organ Failure Assessment, *IAI* intra-abdominal infection, *UTI* urinary tract infection, *MV* mechanical ventilator

**Table 2:** Demographic characteristics at ICU admission (continuous variable)

Variables	Mean ± SD	Range
Age (years)	66.3 ± 14.7	30–87
BMI (kg/m^2^)	23.6 ± 3.6	16.6–30.9
SOFA score	7.9 ± 5.0	1–21
APACHE II score	17.4 ± 8.0	7–38
ICU stay (days)	12.6 ± 25.3	1–103
Hospital stay (days)	39.1 ± 25.1	2–114
Duration of MV (days)	8.9 ± 20.3	1–103
Glasgow Coma Scale	11.6 ± 2.7	3–15
Survival time for expired patients (days)	35.1 ± 33.9	1–103
Albumin (g/dL)	2.7 ± 0.57	1.7–4.2
CRP (mg/L)	123.3 ± 95.8	4.7–311.1
Procalcitonin (ng/mL)	33.8 ± 58.7	0.0–200.0
Lactate (mg/dL)	40.4 ± 40.2	10.0–179.7
Creatinine (mg/dL)	1.4 ± 1.7	0.4–9.7
eGFR (mL/min)	80.4 ± 51.3	5.3–192.0
Na (mEq/L)	138.0 ± 6.3	127.0–157.0
K (mEq/L)	4.0 ± 0.8	2.0–6.0
Bilirubin (mg/dL)	3.6 ± 5.9	0.1–31.0
INR	1.6 ± 0.6	1.1–3.3
Platelet count (103/uL)	166.1 ± 137.0	25.0–613.0

*ICU* intensive care unit, *SD* standard deviation, *BMI* body mass index, *SOFA* Sequential Organ Failure Assessment, *APACHE* Acute Physiology and Chronic Health Evaluation, *MV* mechanical ventilation, *CRP* C-reactive protein, *eGFR* estimated glomerular filtration rate, *INR* international normalized ratio

### Analysis of biomarkers and clinical parameters

**Fig. 2:**
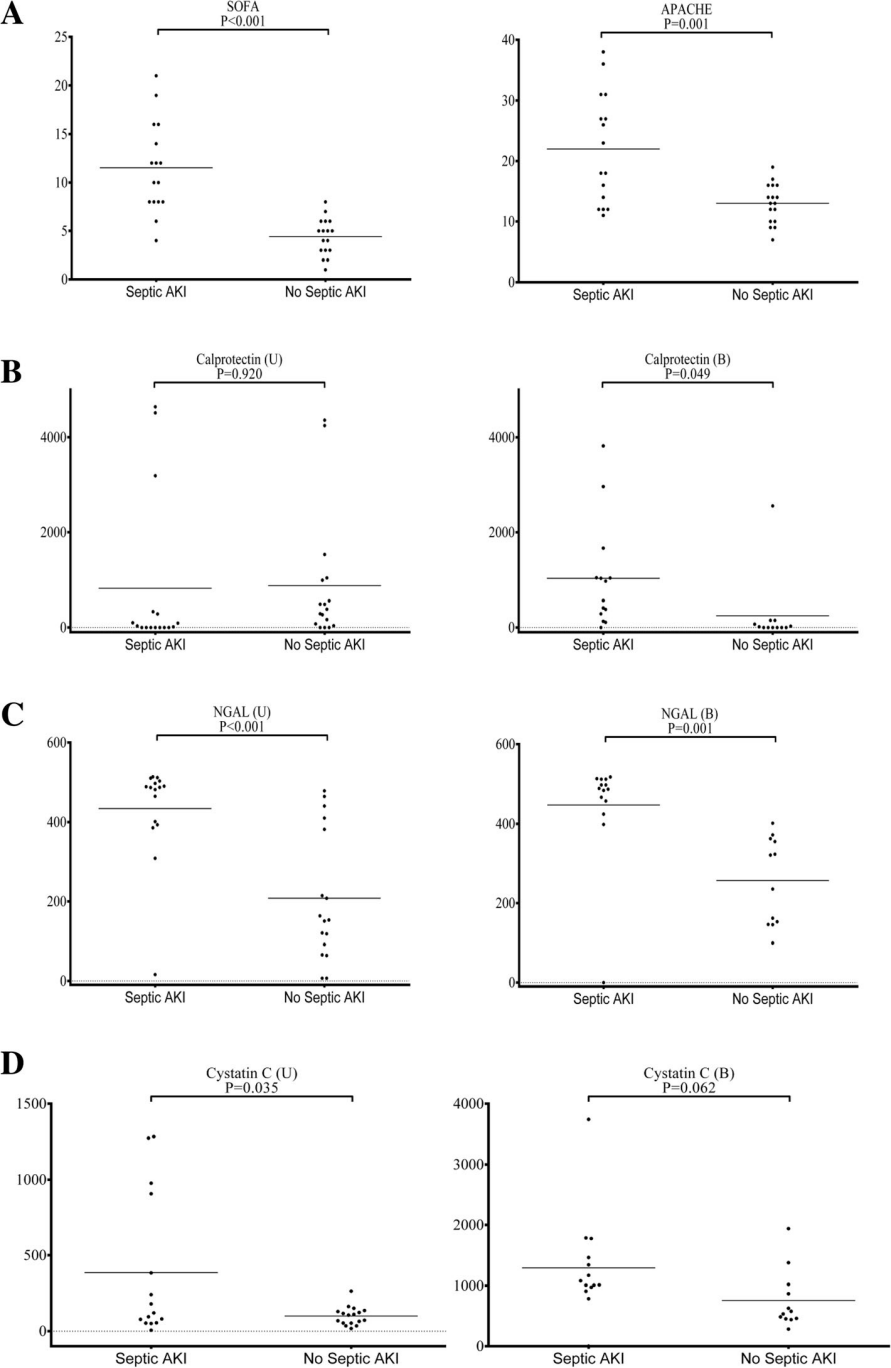
**a**–**d** Septic AKI. SOFA and APACHE II scores and serum and urinary levels of calprotectin, NGAL, and cystatin C. SOFA and APACHE II scores (**a**), calprotectin (**b**), NGAL (**c**), and cystatin C (**d**). The serum and urinary levels are represented as scatter dot plots, and the medians are reported. The arithmetic means of the tested parameters are indicated by a line. SOFA Sequential Organ Failure Assessment, APACHE Acute Physiology and Chronic Health Evaluation, NGAL neutrophil gelatinase-associated lipocalin

**Fig. 3:**
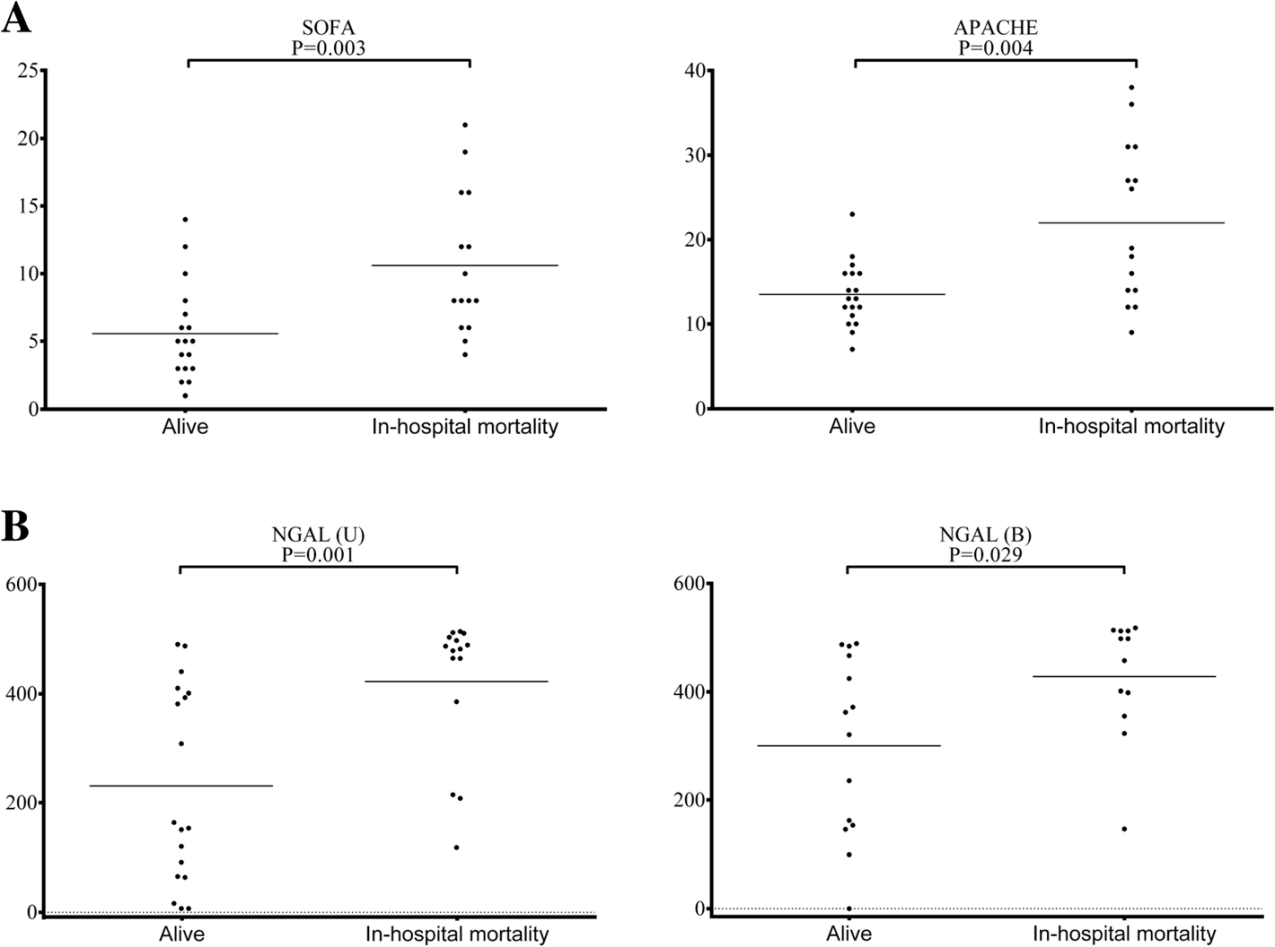
**a**–**b** In-hospital mortality. SOFA and APACHE II scores and serum and urinary levels of NGAL. **a** SOFA and APACHE II scores. **b** NGAL. The serum and urinary levels are represented as scatter dot plots, and the medians are reported. The arithmetic means of the tested parameters are indicated by a line. SOFA Sequential Organ Failure Assessment, APACHE Acute Physiology and Chronic Health Evaluation, NGAL neutrophil gelatinase-associated lipocalin

Serum and urinary levels of calprotectin, NGAL, and cystatin C, in addition to the SOFA and APACHE II scores, were measured in septic AKI patients and represented as scatter dot plots in Fig. 2. Serum and urinary levels of GDF-15 and KIM-1 are depicted in the Additional file 1: Figure S1. Similar scatter dot plots representing in-hospital mortality are shown in Fig. 3 and in the Additional file 2: Figure S2.

### Sepsis

The mean levels of the five different biomarkers, namely, NGAL, calprotectin, KIM-1, cystatin C, and GDF-15, in the serum and urine samples were compared, as shown in the Sepsis column of Table 3. Patients with sepsis had significantly higher levels of both serum and urinary NGAL (both *P* values < 0.001). Serum and urinary cystatin C (*P* value 0.016 and *P* value 0.046, respectively) levels were also significantly higher in septic patients. Other biochemical parameters showing statistical significance included serum albumin, creatinine, and CRP (*P* values 0.025, 0.004, and < 0.001, respectively). Septic patients also had statistically higher SOFA (*P* value 0.001) and APACHE II (*P* value 0.001) scores upon ICU admission.

**Table 3:** Analysis of various biomarkers and clinical parameters in surgical ICU regarding sepsis, septic AKI, and in-hospital mortality

Variables	Sepsis			Septic AKI			In-hospital mortality		
	Yes^a^	No^a^	*P* value	Yes^a^	No^a^	*P* value	Yes^a^	No^a^	*P* value
Serum									
NGAL (ng/mL)	428.1 ± 29.1	204.7 ± 34.2	< 0.001	447.5 ± 35.7	256.5 ± 31.8	0.001	428.2 ± 32.3	300.4 ± 44.3	0.029
Calprotectin (pg/mL)	809.7 ± 251.2	353.3 ± 315.6	0.302	1030.3 ± 298.6	248.1 ± 210.7	0.049	1013.8 ± 354.8	373.9 ± 193.1	0.113
KIM-1 (pg/mL)	694.9 ± 398.8	142.9 ± 289.1	0.392	1150.5 ± 448.5	275.5 ± 135.1	0.081	838.1 ± 500.2	256.8 ± 329.9	0.329
Cystatin C (ng/ml)	1223.1 ± 186.8	640.5 ± 120.9	0.016	1290.4 ± 222.3	756.3 ± 138.9	0.062	1312.8 ± 260.4	813.3 ± 124.4	0.076
GDF-15 (ng/mL)	8.8 ± 2.7	9.5 ± 2.8	0.330	6.7 ± 2.7	10.8 ± 2.7	0.121	6.2 ± 1.9	11.5 ± 3.1	0.322
Urine									
NGAL (ng/mL)	393.2 ± 33.4	167.0 ± 41.9	< 0.001	434.2 ± 31.5	208.3 ± 39.5	< 0.001	422.3 ± 33.7	230.8 ± 42.2	0.001
Calprotectin (pg/mL)	933.4 ± 346.6	690.3 ± 367.5	0.462	825.1 ± 415.2	878.1 ± 328.8	0.102	646.2 ± 340.3	1024.2 ± 384.8	0.190
KIM-1 (pg/mL)	7149.0 ± 1491.0	9649.2 ± 2975.0	0.510	7377.5 ± 1970.0	8551.7 ± 2014.0	0.488	8765.3 ± 1949.0	7329.9 ± 2006.0	0.616
Cystatin C (ng/mL)	315.4 ± 89.9	79.5 ± 11.1	0.046	386.1 ± 121.5	100.4 ± 14.8	0.035	324.5 ± 115.6	154.7 ± 53.4	0.628
GDF-15 (ng/mL)	25.1 ± 3.2	34.0 ± 3.6	0.095	26.2 ± 3.6	29.8 ± 3.7	0.487	29.4 ± 3.8	26.9 ± 3.5	0.642
SOFA score	9.5 ± 1.1	4.6 ± 0.6	0.001	11.5 ± 1.2	4.4 ± 0.5	< 0.001	10.6 ± 1.4	5.6 ± 0.8	0.003
APACHE II score	20.0 ± 1.8	12.1 ± 0.9	0.001	22.0 ± 2.3	13.0 ± 0.8	0.001	22.0 ± 2.4	13.5 ± 0.9	0.004
Albumin (g/dL)	2.6 ± 0.1	3.0 ± 0.1	0.025	2.5 ± 0.1	3.0 ± 0.1	0.007	2.4 ± 0.1	3.0 ± 0.1	0.003
Creatinine (mg/dL)	1.8 ± 0.4	0.7 ± 0.1	0.004	2.2 ± 0.6	0.7 ± 0.1	< 0.001	2.1 ± 0.6	0.9 ± 0.1	0.027
Procalcitonin (ng/mL)^b^	28.8 ± 10.9	0.2	0.095	35.9 ± 14.2	6.2 ± 3.1	0.205	29.6 ± 13.2	25.1 ± 17.3	0.512
Lactate (mg/dL)^b^	40.4 ± 9.2	N.A.	N.A.	47.7 ± 11.9	19.8 ± 3.8	0.190	49.9 ± 15.4	27.3 ± 3.6	0.180
CRP (mg/L)^b^	142.1 ± 20.4	40.9 ± 12.9	< 0.001	163.9 ± 24.7	64.4 ± 15.8	0.008	132.6 ± 24.2	113.3 ± 28.8	0.610
Age (year)	63.1 ± 3.4	72.8 ± 2.8	0.072	64.1 ± 3.9	68.5 ± 3.4	0.397	63.9 ± 4.2	68.3 ± 3.2	0.400
BMI (kg/m^2^)	23.0 ± 0.7	24.7 ± 1.1	0.228	23.0 ± 0.9	24.2 ± 0.8	0.350	22.3 ± 0.9	24.6 ± 0.8	0.066

*AKI* acute kidney injury, *NGAL* neutrophil gelatinase-associated lipocalin, *GDF-15* growth differentiation factor 15, *KIM-1* kidney injury molecule-1, *SOFA* Sequential Organ Failure Assessment, *APACHE* Acute Physiology and Chronic Health Evaluation, *BMI* body mass index, *N.A*. not applicable

^a^Expressed as mean ± standard error of mean (SEM)

^b^Not routinely checked in patients without sepsis

### Septic AKI

As shown in the Septic AKI column of Table 3, the levels of serum and urinary NGAL (*P* values 0.001 and < 0.001, respectively) were significantly higher in patients with septic AKI than in those without. Although serum calprotectin (*P* value 0.049) showed statistical significance, urinary calprotectin (*P* value 0.102) was not significantly elevated in patients with septic AKI. The levels of serum albumin, creatinine, CRP, SOFA score, and the APACHE II score (*P* values 0.007, < 0.001, 0.008, < 0.001, and 0.001, respectively) all showed significant elevation in the septic AKI group when compared to the non-septic AKI group.

### In-hospital mortality

Patients with in-hospital mortality (*n* = 15) showed significantly higher levels of serum NGAL (*P* value 0.029) and urinary NGAL (*P* value 0.001) on ICU admission, as shown in the In-hospital Mortality column of Table 3. The SOFA and APACHE II scores, serum albumin, and creatinine also showed statistical significance (*P* values 0.003, 0.004, 0.003, and 0.027, respectively).

### Performance of SOFA and biomarkers

Table 4 summarizes the area under the ROC curves (AUROC) of significant variables regarding septic AKI and in-hospital mortality. In predicting septic AKI, serum and urinary NGAL showed an AUROC of 0.991 and 0.915, respectively. Serum calprotectin showed an AUROC of 0.889. Other parameters with statistical significance in predicting septic AKI included serum creatinine, CRP, SOFA, and APACHE II scores, with the exception of serum albumin.

**Table 4:** Area under the ROC curve (AUROC) of significant variables regarding septic AKI and in-hospital mortality

Variables	Septic AKI				In-hospital mortality			
	AUROC	*P* value	Cutoff value	Sensitivity/specificity (%)	AUROC	*P* value	Cutoff value	Sensitivity/specificity (%)
Serum NGAL	0.991	< 0.001	413.2	92.3/100	0.768	0.021	385.3	75.0/64.3
Urinary NGAL	0.915	0.001	383.7	92.3/77.8	0.780	0.016	383.7	75.0/64.3
Serum calprotectin	0.889	0.002	219.8	84.6/88.9				
Serum KIM-1	0.752	0.049	17.2	69.2/77.8				
Urinary Cystatin C	0.641	0.271	118.9	53.8/66.7				
Albumin	0.269	0.071	2.6	38.5/33.3	0.232	0.021	2.5	41.7/35.7
Creatinine	0.966	< 0.001	0.9	100.0/88.9	0.676	0.129	0.9	75.0/57.1
CRP	0.795	0.021	82.4	76.9/77.8				
SOFA score	0.957	< 0.001	7.5	92.3/88.9	0.774	0.018	7.5	75.0/71.4
APACHE II Score	0.842	0.008	13.5	76.9/77.8	0.762	0.024	15.0	66.7/71.4
Serum NGAL+ Serum Calprotectin + SOFA score	1.000	< 0.001						
Serum NGAL + urinary NGAL + SOFA score					0.911	< 0.001		

*AKI* acute kidney injury, *NGAL* neutrophil gelatinase-associated lipocalin, *SOFA* Sequential Organ Failure Assessment, *APACHE* Acute Physiology and Chronic Health Evaluation

In predicting in-hospital mortality, serum and urinary NGAL gave a statistical significant AUROC of 0.768 and 0.780, respectively. Similarly, the SOFA and APACHE II scores showed an AUROC of 0.774 and 0.762, respectively. Albumin also showed statistical significance in predicting in-hospital mortality, even though no statistical significance was observed in predicting septic AKI. Of great interest, a combination of serum NGAL, serum calprotectin, and SOFA score presented an AUROC of 1.000 (*P* value < 0.001) for septic AKI, while a combination of serum NGAL, urinary NGAL, and SOFA score gave an AUROC of 0.911 (*P* value < 0.001) for in-hospital mortality.

**Fig. 4:**
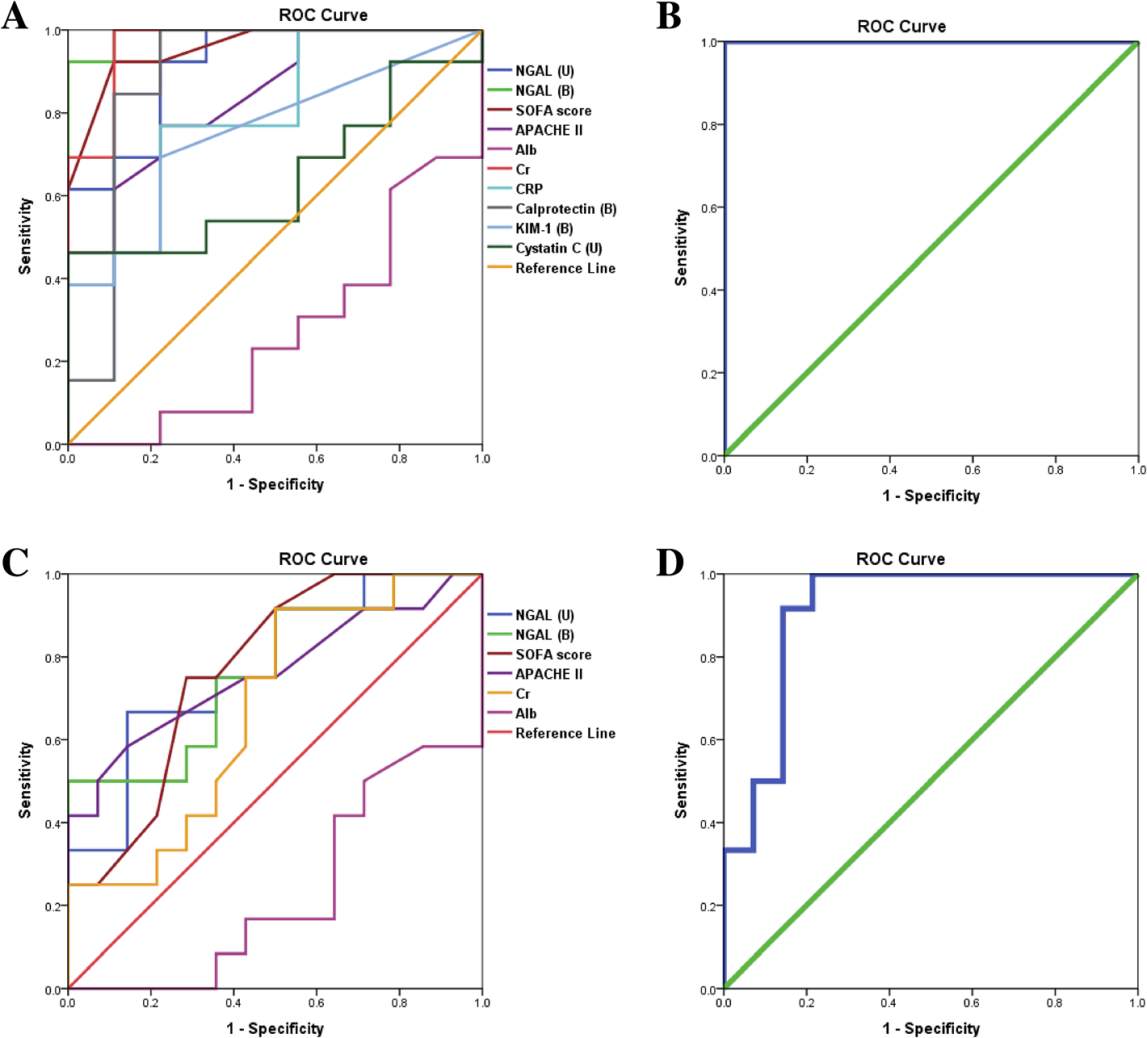
**a**–**d** Performance of SOFA score and biomarkers. **a** ROC curves of significant variables in predicting septic AKI. **b** ROC curve of serum NGAL, serum calprotectin, and SOFA score in predicting septic AKI. **c** ROC curves of significant variables in predicting in-hospital mortality. **d** ROC curve of serum and urinary NGAL and SOFA score in predicting in-hospital mortality. SOFA Sequential Organ Failure Assessment, NGAL neutrophil gelatinase-associated lipocalin

The ROC curves of individual plasma and urinary biomarkers along with the SOFA and APACHE II scores are shown in Fig. 4a, c, for the prediction of septic AKI and in-hospital mortality, and a combination of biomarkers and SOFA score is shown in Fig. 4b, d.

Further analysis was performed within the sepsis cohort, as shown in Additional file 3: Table S1. In predicting septic AKI, serum and urinary NGAL and serum calprotectin appeared to be statistically significant with an AUROC of 0.981, 0.885, and 0.962, respectively. A combination of serum NGAL, serum calprotectin, and SOFA score gave a high AUROC of 1.000 (*P* value 0.003). In predicting in-hospital mortality, similarly to what was demonstrated previously, a combination of serum NGAL, urinary NGAL, and SOFA score gave an AUROC of 0.963 (*P* value 0.001). The ROC curves of a combination of biomarkers and SOFA score in predicting septic AKI and in-hospital mortality are demonstrated in Additional file 4: Figure S3A and B.

## Discussion

The incidence of sepsis remains high among critically ill patients. Septic patients tend to have longer ICU stays, hospital stays, and significantly higher ICU and in-hospital mortality than those in the general ICU population [24]. The new Sepsis-3 criteria include suspected or documented infection and a two-point increase in SOFA score. The first step to optimal treatment of sepsis is to promptly identify patients with sepsis. The SOFA score has been shown to have a high predictive validity and prognostic accuracy for in-hospital mortality, with an AUROC of 0.74 and 0.753, respectively [2, 3]. Because septic AKI is common during the first 24 h after ICU admission and is associated with higher ICU and in-hospital mortality [25], studies on biomarkers have become of great interest. With the recent literature available on novel biomarkers identified in septic AKI, we have conducted a prospective study in a surgical intensive care unit, investigating the use of NGAL, calprotectin, KIM-1, cystatin-C, and GDF-15 in combination with SOFA to improve early recognition of such patients.

NGAL has been proven to be a valuable biomarker for early identification of AKI [26]. Studies have been conducted investigating the predictive value of NGAL as a biomarker of septic AKI. Plasma NGAL has been shown to have an AUC of 0.86, indicating an adequate diagnostic accuracy [11]. Urine NGAL, similarly, showed an AUC of at least 0.84 in predicting septic AKI [27, 28]. NGAL also appears to be an independent predictor of 7-day and 28-day mortality in critically ill patients, with an AUROC of 0.883 and 0.723, respectively [29]. Supported by the literature, we have also demonstrated that plasma and urinary NGAL showed a comparable predictive value for septic AKI and in-hospital mortality in critically ill surgical patients. A combination of serum and urinary NGAL and SOFA score showed a high AUROC of 0.911, providing a better predictor of in-hospital mortality than any single parameter alone.

Calprotectin, a heterodimer complex of S100A8/A9 primarily released by neutrophils, monocytes, and macrophages, lately has been studied extensively [30]. Gao et al. [31] have shown that the level of calprotectin was correlated with the degree of sepsis severity, with an AUROC of 0.901 and a sensitivity and specificity of 83.1% and 88.5%, respectively. Similar to our study, they demonstrated that calprotectin levels were significantly higher in patients with septic AKI and in non-survivors at 28 days than in those not meeting these conditions. We have also successfully revealed calprotectin to be a sensitive and specific biomarker in detecting septic AKI, with an AUROC as high as 0.889.

As sepsis is frequently complicated by AKI, which, in turn, is a major risk factor of mortality, a prompt diagnosis of sepsis is also crucial to establish timely treatment. The present study demonstrated that although the clinical practice scoring systems such as SOFA and APACHE showed good diagnostic and prognostic ability, a panel of serum/urinary NGAL and serum calprotectin and SOFA score raised the AUROC to 1.000 in diagnosing septic AKI and to 0.911 in predicting in-hospital mortality. Further analysis within the sepsis cohort demonstrated that such a panel likewise raised the AUROC to 1.000 in predicting septic AKI and to 0.963 in predicting in-hospital mortality. However, despite such promising results, some limitations apply. First, the sample size was limited, which may have led to patient selection bias. Second, although the results appear promising, only roughly 10% of the ICU patients were enrolled in the study, and the results should be extrapolated cautiously to other critically ill patients. Third, the temporal changes of biomarkers and clinical scores were not obtained. Last but not the least, the current study did not examine the relationship between intra-abdominal pressure and renal function. An elevation in intra-abdominal pressure or intra-abdominal hypertension (IAH) has long been recognized as a risk factor for the development of altered renal function among critically ill patients [32, 33]. IAH has been reported to occur in 51–76% of patients with septic shock and in 33–41% of patients after emergency abdominal surgery and is associated with AKI and mortality [34, 35]. In addition to timely recognition and management of IAH to lower IAP, novel biomarkers may be utilized to predict prognosis in patients with established AKI [36]. As a result, future studies with a larger population size incorporating dynamic changes of biomarkers and intra-abdominal pressure may be warranted. Even though we have shown favorable results using biomarkers and the SOFA score in predicting the development of septic AKI and in-hospital mortality, in daily practice, other more immediate parameters and clinical judgment may be useful in the early assessment of these critically ill patients.

## Conclusions

Septic AKI arises in more than 50% of patients with sepsis, with a six- to eightfold increase in the risk of in-hospital mortality. Thus far, no single scoring system appears sufficiently sensitive and specific in predicting the development of septic AKI and in-hospital mortality for critically ill patients. In this pilot study, we have established a panel incorporating serum biomarkers and the SOFA score that appears promising in the early detection of septic AKI, which, in turn, opens a window for prompt treatment in the clinical setting. Furthermore, the panel presents with a great prognostic value for in-hospital mortality among patients in surgical intensive care units. That said, further, larger well-designed studies are warranted.

## Additional files

Additional file 1: **Figure S1.** (A–B) Septic AKI. Serum and urinary levels of GDF-15 and KIM-1 measured by ELISA in patients with or without septic AKI. The serum and urinary levels are represented as scatter dot plots, and the medians are reported. The arithmetic means of the tested parameters are indicated by a line. (A) GDF-15; (B) KIM-1. B in parenthesis indicates blood samples; U in parenthesis indicates urine samples. GDF-15 growth differentiation factor 15, KIM-1 Kidney Injury Molecule-1. (TIF 77 kb)

Additional file 2: **Figure S2.** (A–D) In-hospital mortality. Serum and urinary levels of calprotectin, Cystatin C, GDF-15, and KIM-1 measured by ELISA in patients with or without in-hospital mortality. The serum and urinary levels are represented as scatter dot plots, and the medians are reported. The arithmetic means of the tested parameters are indicated by a line. (A) Calprotectin, (B) cystatin C, (C) GDF-15, and (D) KIM-1. B in parenthesis indicates blood samples; U in parenthesis indicates urine samples. GDF-15 growth differentiation factor 15, KIM-1, Kidney Injury Molecule-1. (TIF 149 kb)

Additional file 3: **Table S1.** Area under the ROC curve (AUROC) of various variables regarding septic AKI and in-hospital mortality within the septic cohort. (DOCX 19 kb)

Additional file 4: **Figure S3. **(A–B). Performance of SOFA score and biomarkers within the septic cohort. (A) ROC curve of serum NGAL, calprotectin, and SOFA score in predicting septic AKI. (B) ROC curve of serum and urinary NGAL and SOFA score in predicting in-hospital mortality. SOFA Sequential Organ Failure Assessment, NGAL neutrophil gelatinase-associated lipocalin. (TIF 1447 kb)

## Abbreviations

ADQI: Acute Dialysis Quality Initiative
AKI: Acute kidney injury
APACHE: Acute Physiology and Chronic Health Evaluation
AUROC: Area under the ROC curve
BMI: Body mass index
CKD: Chronic kidney disease
CRP: C-Reactive protein
eGFR: Estimated glomerular filtration rate
ELISA: Enzyme-linked immunosorbent assay
ESRD: End-stage renal disease
FiO_2_: Fraction of inspired oxygen
GCS: Glasgow Coma Scale
GDF-15: Growth differentiation factor 15
GFR: Glomerular filtration rate
IAI: Intra-abdominal infection
ICU: Intensive care unit
INR: International normalized ratio
IRB: Institutional review board
KIM-1: Kidney injury molecule-1
MAP: Mean arterial blood pressure
MV: Mechanical ventilation
NGAL: Neutrophil gelatinase-associated lipocalin
PaO_2_: Partial pressure of oxygen
RIFLE: Risk, Injury, Failure, Loss, and End-Stage Renal Disease
ROC Curve: Receiver operating characteristic curve
sCr: Serum creatinine
SD: Standard deviation
SOFA: Sequential Organ Failure Assessment
UTI: Urinary tract infection
