# Supplementary material for: A combination of SOFA score and biomarkers gives a better prediction of septic AKI and in-hospital mortality in critically ill surgical patients: a pilot study
Source: World J Emerg Surg. 2018 Sep 10;13:41. doi: 10.1186/s13017-018-0202-5 (PMC6131912; doi:10.1186/s13017-018-0202-5)
Supplement: Supplementary file 3 — Table S1. Area under the ROC curve (AUROC) of various variables regarding septic AKI and in-hospital mortality within the septic cohort. (DOCX 19 kb) [file 13017_2018_202_MOESM3_ESM.docx]

**Additional file 3: Table S1 –Area under the ROC curve (AUROC) of various variables regarding septic AKI and in-hospital mortality within the septic cohort**

| **Variables** |  | | **Septic AKI** | | | | |  | **In-hospital mortality** | | | |
| --- | --- | --- | --- | --- | --- | --- | --- | --- | --- | --- | --- | --- |
|  |  | | **AUROC** | | ***P*-value** | **Cutoff**  **value** | **Sensitivity/**  **Specificity(%)** |  | **AUROC** | ***P­*-value** | **Cutoff**  **value** | **Sensitivity/**  **Specificity(%)** |
| **Serum NGAL**  **Urinary NGAL** | |  | | 0.981 | 0.005  0.024 | 413.2  383.7 | 92.3/100  92.3/75.0 |  | 0.750  0.775 | 0.076  0.051 | 441.2  383.7 | 70.0/50.0  90.0/50.0 |
|  |  | 0.885 | | |  |  |  |  |  |  |  |  |
| **Serum calprotectin** | | 0.962 | | | 0.007 | 219.8 | 84.6/100 |  |  |  |  | |
| **Serum KIM-1** | | 0.846 | | | 0.042 | 17.2 | 69.2/100 |  |  |  |  | |
| **Urinary Cystatin C** | | 0.596 | | | 0.147 | 115.7 | 53.8/50.0 |  |  |  |  | |
| **Albumin** | | 0.433 | | | 0.692 | 2.495 | 38.5/50.0 |  | 0.244 | 0.069 | 2.495 | 30.0/50.0 |
| **Creatinine** | | 0.923 | | | 0.013 | 0.875 | 100/75.0 |  | 0.631 | 0.351 | 0.930 | 90.0/37.5 |
| **CRP** | | 0.654 | | | 0.365 | 82.35 | 76.9/50.0 |  |  |  |  | |
| **SOFA score** | | 0.990 | | | 0.004 | 7.0 | 92.3/100 |  | 0.700 | 0.155 | 7.0 | 80.0/50.0 |
| **APACHE II Score** | | 0.740 | | | 0.157 | 13.0 | 76.9/50 |  | 0.794 | 0.037 | 13.0 | 80.0/50.0 |
| **Serum NGAL+ serum**  **calprotectin + SOFA**  **score** | | 1.000 | | | 0.003 |  |  |  |  |  |  | |
| **Serum NGAL + urinary**  **NGAL + SOFA score** | |  | | |  |  |  |  | 0.963 | 0.001 |  | |

AKI, acute kidney injury; NGAL, neutrophil gelatinase-associated lipocalin; SOFA, sequential organ failure assessment; APACHE, acute physiology and chronic health evaluation
