# Supplementary figures and images for: A combination of SOFA score and biomarkers gives a better prediction of septic AKI and in-hospital mortality in critically ill surgical patients: a pilot study
Source: World J Emerg Surg. 2018 Sep 10;13:41. doi: 10.1186/s13017-018-0202-5 (PMC6131912; doi:10.1186/s13017-018-0202-5)

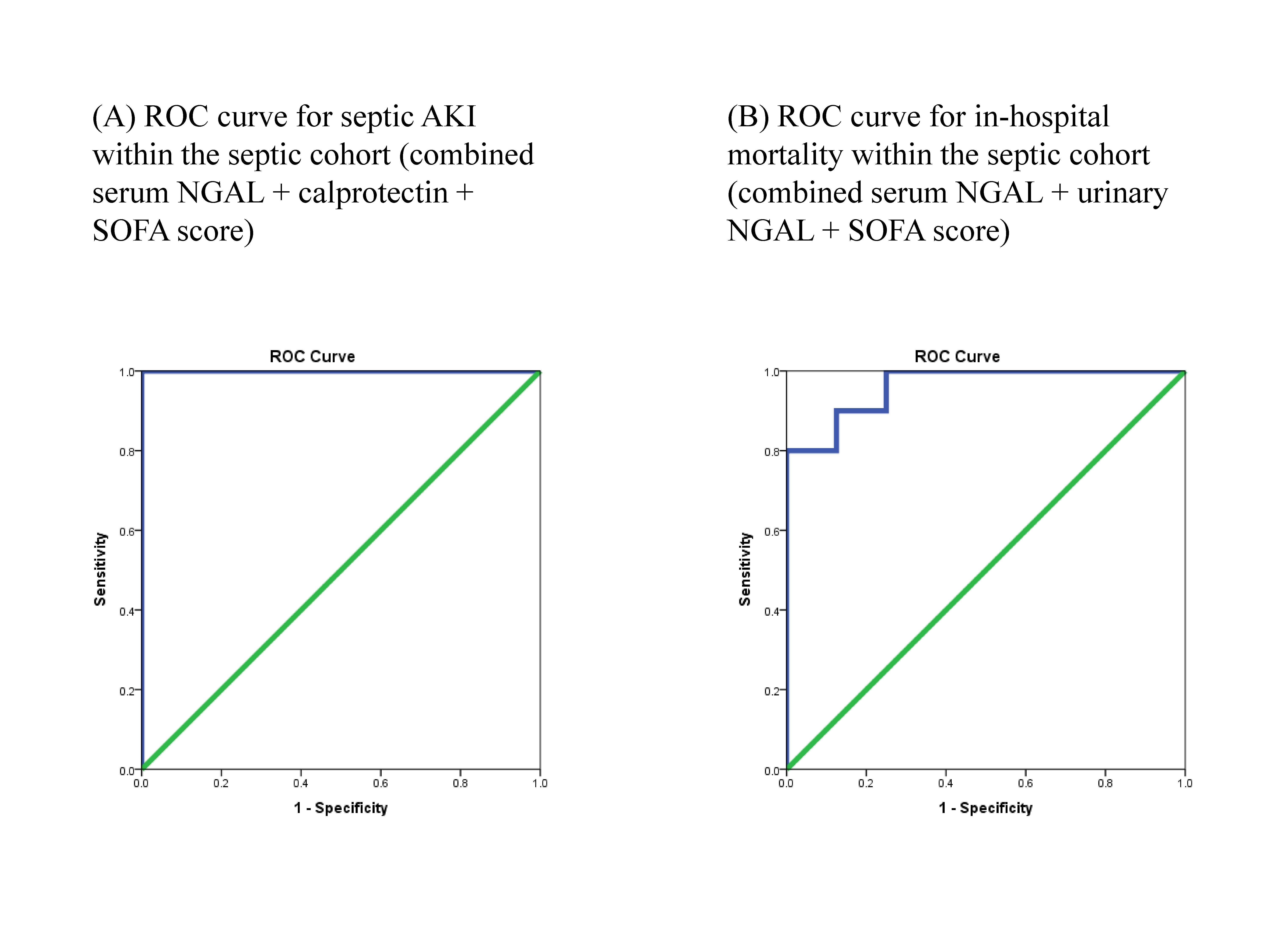

Supplement: Supplementary file 4 — Figure S3. (A–B). Performance of SOFA score and biomarkers within the septic cohort. (A) ROC curve of serum NGAL, calprotectin, and SOFA score in predicting septic AKI. (B) ROC curve of serum and urinary NGAL and SOFA score in predicting in-hospital mortality. SOFA Sequential Organ Failure Assessment, NGAL neutrophil gelatinase-associated lipocalin. (TIF 1447 kb) [file 13017_2018_202_MOESM4_ESM.tif]
